# Hemoglobin Variant (Hemoglobin Aalborg) Mimicking Interstitial Pulmonary Disease

**DOI:** 10.1155/2014/701839

**Published:** 2014-10-21

**Authors:** Vasiliki Panou, Peter-Diedrich Mathias Jensen, Jan Freddy Pedersen, Lars Pilegaard Thomsen, Ulla Møller Weinreich

**Affiliations:** ^1^Department of Respiratory Diseases, Aalborg University Hospital, Mølleparkvej 4, 9000 Aalborg, Denmark; ^2^Department of Haematology, Aalborg University Hospital, Denmark; ^3^Department of Clinical Biochemistry, Aalborg University Hospital, Denmark; ^4^Respiratory and Critical Care Group (RCARE), Centre for Model Based Medical Decision Support Systems, Department of Health Science and Technology, Aalborg University, Denmark

## Abstract

Hemoglobin Aalborg is a moderately unstable hemoglobin variant with no affiliation to serious hematological abnormality or major clinical symptoms under normal circumstances. Our index person was a healthy woman of 58, not previously diagnosed with hemoglobinopathy Aalborg, who developed acute respiratory failure after a routine cholecystectomy. Initially she was suspected of idiopathic interstitial lung disease, yet a series of tests uncovered various abnormal physiological parameters and set the diagnosis of hemoglobinopathy Aalborg. This led us to examine a group of the index person's relatives known with hemoglobinopathy Aalborg in order to study whether the same physiological abnormalities would be reencountered. They were all subjected to spirometry and body plethysmography, six-minute walking test, pulse oximetry, and arterial blood gas samples before and after the walking test. The entire study population presented the same physiological anomalies: reduction in diffusion capacity, and abnormalities in P_a_O_2_ and p50 values; the latter could not be presented by the arterial blood gas analyzer; furthermore there was concordance between pulse oximetry and arterial blood gas samples regarding saturation. These data suggest that, based upon the above mentioned anomalies in physiological parameters, the diagnosis of hemoglobinopathy Aalborg should be considered.

## 1. Introduction

Unstable hemoglobinopathies (Hb) are a rare disease entity of mutational events in the hemoglobin which are characterized by substitutions in the primary sequence of the globin [1]. These mutations alter the tertiary or quaternary structure of the molecule and therefore cause destabilization of the hemoglobin tetramer. A wide range of hemoglobin instabilities are known from in vitro studies, and the clinical findings span from subclinical cases to cases with severe hemolytic disease, for example, beta-thalassemia major or sickle cell disease [2]. Hb present with a broad spectrum of clinical manifestations; however, patients often have very few symptoms apart from slight fatigue, because the hemoglobin is often stable under clinically stable conditions [1–4].

Hemoglobin Aalborg is a rare, unstable hemoglobin variant where a glycine residue (E18) is replaced by arginine (*β*74(E18)Gly->Arg). The incidence and prevalence of Hb Aalborg are not known. It has a reduced oxygen affinity, both in the absence and in the presence of organic phosphates, and a raised oxygen affinity for organic phosphates, despite the fact that the replaced amino acid residue is too far from the heme to affect it directly. The reduced oxygen affinity of the hemoglobin results from constraints on the deformability of heme T-state, which disable oxygen binding and spontaneous conversion from the T-state to the R-state in the absence of oxygen [2, 5].

Hb Aalborg is considered moderately unstable and it is not associated with any severe hematological abnormality. It may cause mild anaemia and Heinz bodies are inducible in the hemoglobin. The vast majority of patients have no or mild symptoms such as fatigue or mild dyspnea on exertion. However, conditions which stress the unstable hemoglobin, such as increased temperature or exposure to oxidant drugs, are likely to precipitate hemoglobin denaturation and significant hemolysis may occur [2, 5].

The index person of the study population was not previously diagnosed with Hb Aalborg and had no underlying chronic disease. However, acute respiratory failure occurred postoperatively in connection to an elective cholecystectomy and no cause of the acute change could be found. As the pulmonary symptoms persisted, idiopathic interstitial lung disease was suspected. The patient was subjected to thorough examination in order to elucidate the cause of the clinical symptoms. Physiological abnormalities, regarding both arterial blood samples analysis and pulse oximetry and body box examination, were observed during the diagnostic procedure.

After the diagnosis of Hb Aalborg was established, a group of family members to the index person, already diagnosed with Hb Aalborg, were also examined and underwent the same tests in order to validate the following hypothesis: “*When anomalies in parameters regarding arterial blood samples analysis, pulse oximetry, and body box examination are encountered in stable state as well as on exertion, Hb Aalborg should be included in the possible, although rare, differential diagnoses.” *The aim of this study was therefore to investigate whether similar abnormalities in the physiological parameters could be detected in all family members. This was described by examining the following:

- diffusion capacity of the lung for carbon monoxide (DLCO),
- the Medical Research Council (MRC) dyspnea score,
- 6-minute walking test,
- oxygen saturation at rest as well as after exertion.

## 2. Materials and Methods

In this prospective study a total of seven people were examined, who all shared the following characteristics: the patients were all heterozygote for Hb Aalborg; six were first degree relatives to the index person and did not have any underlying pulmonary disease. The group consisted of two men and four women, aged 19–82. Two of the subjects were current smokers: patient number 2 with 13 pack-years and currently smoking 10 cigarettes per day and patient number 6 with 2.5 pack-years, currently smoking 20 cigarettes per day. The remaining four persons were never smokers. None of the participants had either significant comorbidities or respiratory complaints. Fatigue, following light physical activity, was described as the only symptom from the entire study population.

All the subjects were examined in the outpatient clinic of the Respiratory Department of Aalborg University Hospital. They were all subjected to spirometry and body plethysmography and six-minute walking test according to guidelines of the American Thoracic Society [6, 7]. The primary goal of the body plethysmography was to obtain accurate DLCO values but the static gas volumes were also measured [8]. Oxygen saturation (s_p_O_2_) was measured by pulse oximetry with Oximax NPB-40, Nellcor, and arterial blood gas samples were taken from arteria radialis by experienced staff, before and after the walking test, according to guidelines [9]. The arterial blood gas samples were analyzed in ABL800 FLEX Analyzer (Radiometer, Copenhagen, Denmark) in order to examine the partial oxygen pressure (p_a_O_2_), carbon dioxide tension (p_a_CO_2_), oxygen saturation fraction (s_a_O_2_), and total hemoglobin (ctHb). Subsequently, functional hemoglobin (ceHb) and the oxygen tension at 50% saturated hemoglobin (p50) were calculated, using the Oxygen Status Algorithm (OSA) [10].

Only descriptive statistics were performed, using Excel.

Prior to the examinations the patients were recruited according to the Helsinki Declaration and signed written consent after oral and written information about the study [11]. The project was presented to the Local Science Ethics Committee of the Region of North Jutland, Denmark, who found no need for ethical review.

## 3. Results

The results of the spirometry, body plethysmography, and 6 minutes' walking test are presented in Table 1. The patients had a median forced expiratory volume in 1 second of percent expected (FEV_1_) of 106% (range 87–128), a median forced vital capacity of percent expected (FVC) of 107% (range 98–134), and a median FEV_1_/FVC ratio of 87 (range 65–99). The median DLCO was 69% (range 59–80) and the median walking distance was 480 m (range 273–568). All the subjects presented with FEV_1_, FVC, and FEV_1_/FVC ratio within the normal range but DLCO was more or less reduced in all the patients with Hb Aalborg. The static gas volumes were found within the normal range for all the subjects (data not shown).

The measurements of pulse oximetry and the results of the arterial blood gas analyses before and after exercise are presented in Table 2. Prior to the walking test, the patients had a median oxygen saturation (s_p_O_2_) of 83% (range 69–87) and oxygen saturation fraction of percent (s_a_O_2_) of 83.3% (range 69.4–87.3), a median of p_a_O_2_ of 91.5 mmHg (range 81–114.8), a median of p50 of 56.85 mmHg (range 45.23–77.25), a median of hemoglobin of 0.74 g/dL (range 0.65–0.82), a median of functional hemoglobin of 0.71 *μ*g/dL (range 0.63–0.76), a median of carboxyhemoglobin of 0.0033 *μ*g/dL (range 0.0015–0.0051), and a median of lactate of 8.11 *μ*g/dL (range 5.41–15.3). After the walking test, a median s_p_O_2_ of 83% (range 81–87) and s_a_O_2_ of 83% (range 81.5–87.5), a median of P_a_O_2_ of 96 mmHg (range 91.5–112.5), a median of p50 of 57.9 mmHg (range 47.85–68.85), a median of hemoglobin of 0.74 g/dL (range 0.69–0.82), a median of functional hemoglobin of 0.72 *μ*g/dL (range 0.62–0.77), a median of carboxyhemoglobin of 0.0036 *μ*g/dL (range 0.0028–0.0043), and a median of lactate of 9 *μ*g/dL (range 6.31–15.32) were observed. Carboxyhemoglobin levels were found in normal range or slightly increased for all patients but two. After the walking test an increase in s_a_O_2_ and P_a_O_2_ was noticed in four subjects and a decrease was seen in two subjects. P50 was elevated both before and after exertion. It was increased in three subjects and decreased in four subjects following exercise.

## 4. Discussion

In this group of patients with Hb Aalborg the common physiological features were the low DLCO, the inability of the blood gas analyzer to present the p50 and p_a_O_2_ values, and low and comparable s_p_O_2_ and s_a_O_2_ levels in contrast to the high partial pressure of oxygen.

No obvious impact of exertion on the hemoglobin was seen. Elevated p50 and low saturation levels in the presence of normal partial oxygen pressure were observed both prior to and after the 6 minutes' walking test. As such our data does not suggest that exertion has an influence on the increase or decrease of these values.

**Figure 1:**
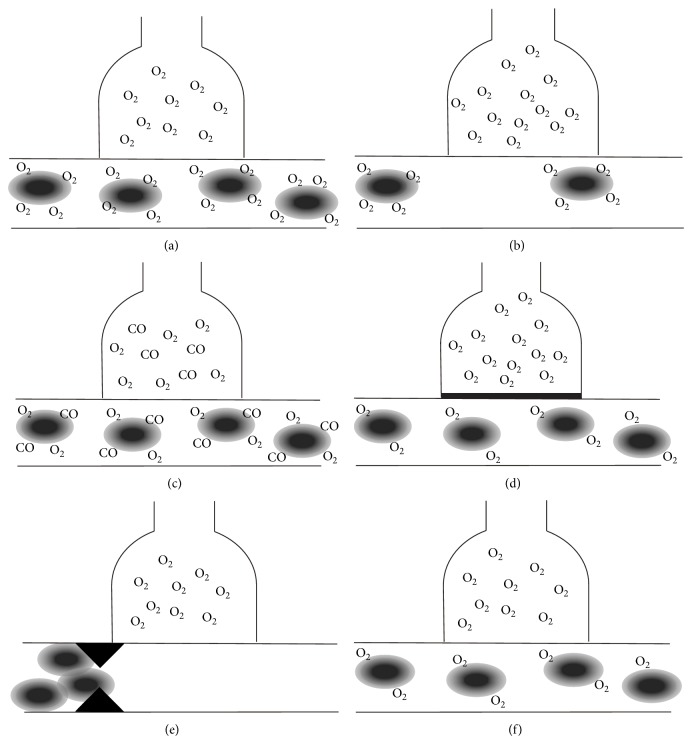
Explanations for reduction in DLCO: (a) normal physiology, (b) anemia, (c) high carboxyhemoglobin levels, (d) pulmonary disease (thickening of the alveolar membrane), (e) pulmonary vascular disease, and (f) Hb Aalborg.

DLCO may be compromised in different compartments. The oxygen supply in the alveoli may be impaired, the diffusion barrier may be the obstacle, or the blood may lack the ability to carry the oxygen to the tissues. The pathology behind this is diverse; it may be caused by anemia, interstitial lung disease, pulmonary vascular disease, increased carboxyhemoglobin, and low oxygen levels in blood [12].  Figure 1 demonstrates how anemia (Figure 1(b)), increased carboxyhemoglobin levels (Figure 1(c)), interstitial lung disease (Figure 1(d)), which results in thickening of the alveolar membrane, and pulmonary vascular disease (Figure 1(e)) affect DLCO. However, the ability of the heme to bind the oxygen is often forgotten in this context (Figure 1(f)).

Reduced DLCO is previously described in the literature in connection with other Hb, such as sickle cell Hb and beta-thalassemia, Hb Canebiére, and Hb Louisville [13–16]. In sickle cell Hb and beta-thalassemia, DLCO is primarily reduced because of pathology in the pulmonary vascular component because of the rigidity of the hemoglobin resulting in microembolism (Figure 1(e)). With regard to beta-thalassemia, iron deposition in the lung tissue, subclinical heart failure as a result of multiple transfusions coupled with a damaged myocardium caused by iron deposition, hepatosplenomegaly and insufficient anatomical and functional development of the lung during early infancy could also alter DLCO [17, 18]. Hb Canebiére and Hb Louisville are Hb with low oxygen affinity, similar to Hb Aalborg (Figure 1(f)) [15, 16]. The hematological changes of Hb Aalborg have been described years ago, but this is, to the authors' knowledge, the first paper to describe the effect of the condition on DLCO. In this study population no clinical explanation to the reduced DLCO was found apart from the unstable Hb Aalborg. None of the subjects in the study population were suspected to have any kind of chronic lung disease, as pulmonary comorbidity was an exclusion criterion.

Hemoglobin and p_a_O_2_ obtained from the arterial blood samples were normal or close to normal in the entire study population. However, the functional hemoglobin of the study objects, which was subsequently calculated, was below the normal levels, as expected for their age, origin, and sex. This suggests that reduced DLCO is a result of the reduced quantity of the functional hemoglobin (Figure 1(f)), which is often combined with a normal hematocrit. Therefore it is essential to consider Hb, which do not affect the hematocrit but the structure of the hemoglobin, as a potential differential diagnosis to conditions where reduced DLCO is present.

The blood gas analyzer would not present p50 values, and p_a_O_2_ values could only be registered manually whilst the blood gas sample was being analyzed. This is due to the analyzer interpreting the simultaneous existence of normal partial pressure of oxygen and low saturation levels as a technical error (error number 1010). P50 is in the context of Hb an interesting parameter. It demonstrates the oxygen tension when 50% of the blood is oxygenated and reflects the hemoglobin's affinity for oxygen. High levels of p50 suggest that high oxygen pressure is required in order to achieve sufficient oxygen supply to the hemoglobin [19]. Increased p50 values have previously been described in Hb with low oxygen affinity, including Hb Aalborg [2, 5, 20]. However, missing data from the arterial blood gas analysis from patients with Hb Aalborg has never been reported previously. The calculated p50 values were all found to be increased.

The discordance between the high p_a_O_2_ and low s_a_O_2_ values is well known in patients with Hb Aalborg [2, 5]. There are several articles describing the presence of low saturation levels in Hb with low oxygen affinity, a finding that sometimes triggers further evaluation leading to identification of an underlying hemoglobin variant [21, 22]. The findings of this study are consistent with those of previous studies. It is, however, noticeable that, in six out of seven subjects, the exact saturation values obtained by pulse oximetry were also verified with accuracy by the blood gas sample analysis despite the low saturation levels and the already diagnosed Hb. Previous studies have demonstrated an overestimation of saturation levels when measured by pulse oximetry compared to those measured by arterial blood gas test at a saturation below 90%–92% [23, 24]. Several factors influence the accuracy of pulse oximetry in conditions of low oxygen saturation, for example, lack of reliable human calibration data during hypoxia and an increased proportion of reduced hemoglobin in hypoxic states, which can exacerbate the error of the absorption ratio [23, 24]. Whether our finding was random or represents another characteristic of Hb Aalborg remains to be clarified by future research.

A slightly decreased ratio between FEV_1_ and FVC is noticed in patient number 3 but the flow-volume curve elucidates that it is due to the subject's poor technique and is not consistent with an actual obstructive pulmonary disease. Carboxyhemoglobin levels were increased in the two patients who were heavy smokers. These patients have, despite male sex and young age, low functional hemoglobin. This is possibly due to the combination of two conditions compromising oxygen DLCO: high carboxyhemoglobin levels and affected oxygen affinity of the heme (Figures 1(c) and 1(f)). Hence it is essential to stress the consequences of smoking to patients with Hb Aalborg. The index person presents a reduced walking distance at the six minutes' walking test. The index person is also the patient with the most prominent symptoms and this reflects that the patient's hemoglobin is under condition of oxidative stress.

The participants are all members of the same family which is a limitation to this study. As such the possibility of a rare variant of Hb Aalborg with distinctive characteristics cannot be excluded. However electrophoresis of the hemoglobin confirms the features well known for Hb Aalborg.

This study describes the deductive use of test results. The above described parameter abnormalities were present in all the group members' tests on a larger or smaller scale. As a consequence, whenever p_a_O_2_ and p50 values cannot be measured in arterial blood gas analysis, a moderately unstable Hb, such as Hb Aalborg, should be considered a possible differential diagnosis. Furthermore, the presence of an unstable Hb should be taken into consideration when reduced DLCO of unknown origin and missing p_a_O_2_ and p50 values from arterial blood gas samples are encountered.

## Figures and Tables

**Table 1:** Results of spirometry and body plethysmography, demonstrating forced expiratory volume in the 1st second in percent of expected value (FEV_1_%), forced ventilatory capacity in percent of expected value (FVC%), the ratio between FEV_1_ and FVC, and diffusing capacity of the lung for carbon monoxide in percent of expected value (DLCO%) and 6-minute walking tests in 7 patients with Hb Aalborg.

	Patient 1 (woman)	Patient 2 (man)	Patient 3 (woman)	Patient 4 (woman)	Patient 5 (woman)	Patient 6 (man)	Index person
FEV_1_ (%)	106	128	94	87	117	106	117
FVC (%)	108	122	103	107	104	98	134
FEV_1_/FVC	87	78	65	94	99	91	74
DLCO (%)	59	69	67	69	76	80	66
Walking distance (m)	417	480	562	420	568	551	273

**Table 2:** Oxygen saturation, measured by pulse oximetry (s_p_O_2_), and results from arterial blood gas analyses: arterial oxygen saturation (SaO_2_), partial pressure of oxygen (PaO_2_), oxygen tension at 50% saturated hemoglobin (P50), and hemoglobin, functional hemoglobin, and lactate concentration before and after 6-minute walking test in 7 patients with Hb Aalborg.

	Patient 1 (woman)	Patient 2 (man)	Patient 3 (woman)	Patient 4 (woman)	Patient 5 (woman)	Patient 6 (man)	Index person
Before exercise							
s_p_O_2 _(%)	83	79	85	73	85	69	87
SaO_2_ (%)	83.3	79	85.5	73.2	85	69.4	87.3
PaO_2_ (mmHg)	91.5	89.25	90.75	81	114.8	100.5	102.75
P50 (mmHg)	59.6	56.85	50.63	45.23	69.68	77.25	54.9
Hemoglobin (g/dL)	0.73	0.82	0.78	0.72	0.65	0.74	0.75
Functional hemoglobin (g/dL)	0.69	0.76	0.74	0.68	0.63	0.71	0.71
Carboxyhemoglobin (g/dL)	0.0037	0.0051	0.003	0.003	0.0015	0.005	0.0033
Lactate (*μ*g/dL)	8.11	8.11	5.41	5.41	15.3	9.91	9.91

After exercise							
s_p_O_2 _(%)	87	81	81	83	85	83	83
SaO_2_ (%)	87.5	81.7	81.5	83.2	85	83	82.9
PaO_2_ (mmHg)	91.5	96	93.75	102.75	91.5	112.5	96.75
P50 (mmHg)	47.85	58.8	57.9	61.28	51.68	68.85	54.23
Hemoglobin (g/dL)	0.73	0.82	0.78	0.74	0.69	0.74	0.77
Functional hemoglobin (g/dL)	0.69	0.77	0.74	0.70	0.62	0.72	0.73
Carboxyhemoglobin (g/dL)	0.0037	0.0043	0.0028	0.0036	0.003	0.0043	0.003
Lactate (*μ*g/dL)	15.32	8.11	6.31	9.91	7.21	9.91	9
